# Supplementary material for: 2-Hydroxypropyl-β-cyclodextrin Regulates the Epithelial to Mesenchymal Transition in Breast Cancer Cells by Modulating Cholesterol Homeostasis and Endoplasmic Reticulum Stress
Source: Metabolites. 2021 Aug 23;11(8):562. doi: 10.3390/metabo11080562 (PMC8399758; doi:10.3390/metabo11080562)
Supplement: Supplementary file 1 [file metabolites-11-00562-s001.zip › metabolites-1265510-supp revised-final proofed.pdf]

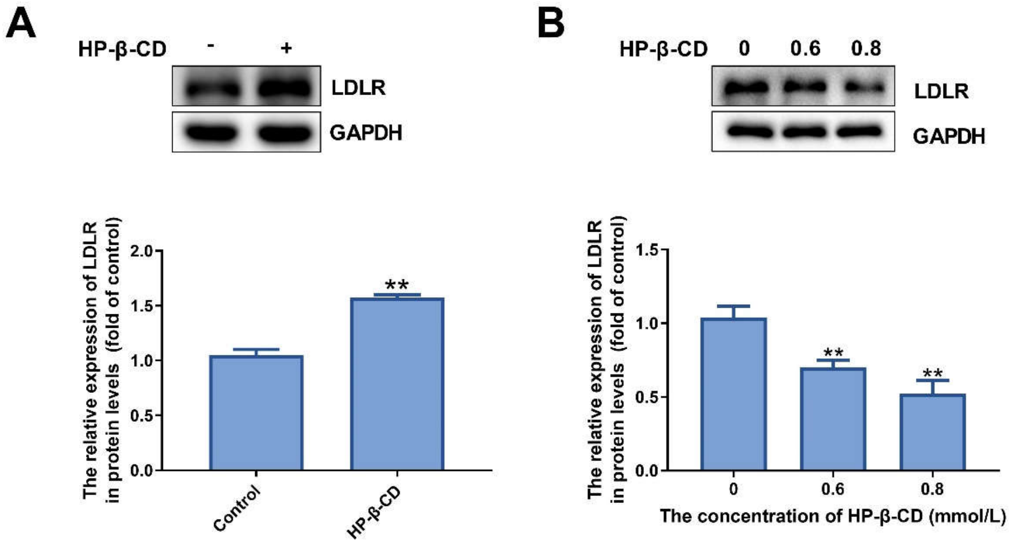

Figure S1. The effect of different concentrations of HP-β-CD for the cholesterol intake in MDA-MB-231 cells. (A) MDA-MB-231 cells were treated with or without 5 mmol/L HP-β-CD. Expression level of LDLR. (B) MDA-MB-231 cells were treated with various concentrations of HP-β-CD (0, 0.6, 0.8 mmol/L). Expression level of LDLR. Data are presented as the mean ± SD (n=3). **P < 0.01 versus the control group.

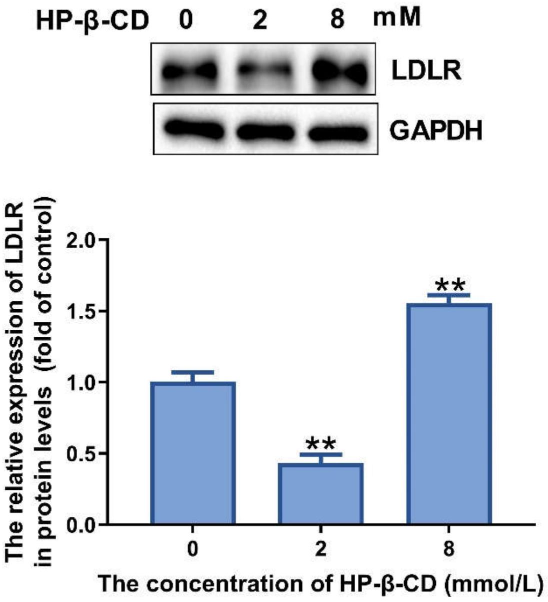

Figure S2. The effect of different concentrations of HP-β-CD for the cholesterol intake in BT-549 cells. BT-549 cells were treated with various concentrations of HP-β-CD (0, 2, 8 mmol/L). Expression level of LDLR. Data are presented as the mean ± SD (n=3). **P < 0.01 versus the control group.
